# Graveoline Analogs Exhibiting Selective Acetylcholinesterase Inhibitory Activity as Potential Lead Compounds for the Treatment of Alzheimer’s Disease

**DOI:** 10.3390/molecules21020132

**Published:** 2016-01-22

**Authors:** Zeng Li, Chaoyu Mu, Bin Wang, Juan Jin

**Affiliations:** 1 College of Pharmacy, Anhui Medical University, Hefei 230032, China; cym19841107@sina.com (C.M.); aywanganyong@163.com (J.J.); 2 Department of Clinical Laboratory, Huaibei Miner’s General Hospital, Huaibei 235000, China; 3 School of Pharmaceutical Sciences, Xiangnan University, Chenzhou 423000, China; aymumu@sohu.com

**Keywords:** graveoline analogs, acetylcholinesterase (AChE), butyrylcholine esterase (BuChE), structure–function relationships (SARs), mechanism

## Abstract

This study designed and synthesized a series of new graveoline analogs on the basis of the structural characteristics of acetylcholinesterase (AChE) dual-site inhibitors. The activity of these analogs was also evaluated. Results showed that the synthesized graveoline analogs displayed stronger inhibitory activity against AChE and higher selectivity than butyrylcholine esterase (BuChE) (Selectivity Index from 45 to 486). When the two sites in the graveoline parent ring substituting phenyl and amino terminal had six chemical bonds (*n* = 3) and the terminal amino was piperidine, compound **5c** showed the best activity. Furthermore, the mechanism of action and binding mode were explored by enzyme kinetic simulation, molecular docking, and thioflavin T-based fluorometric assay. Cytotoxicity assay showed that the low concentration of the analogs did not affect the viability of the neurocyte SH-SY5Y.

## 1. Introduction

Alzheimer’s disease (AD) is a chronic progressive degenerative disease of the central nervous system [1]. The degeneration of the cerebral cortex leads to the loss of its normal function, including memory, judgment, abstract thinking ability, reasoning ability, and spatial relationship, thus resulting in the complete loss of a patient’s self-help ability [2]. This disease is a primary cerebral disease. The onset of AD occurs slowly and is mostly affected by family history. Furthermore, AD is progressive and is the most common cause of dementia among the elderly [3].

AD pathogenesis has not yet been fully elucidated, and many hypotheses on AD pathogenesis have been developed. Among them, the brain cholinergic neuron damage hypothesis and amyloid-β (Aβ) cascade hypothesis are widely recognized; that is, brain cholinergic neuron damage and Aβ cascade are speculated to be the dominant causes of AD pathogenesis [4,5,6]. The acetylcholinesterase (AChE) inhibitor, based on the cholinergic hypothesis, is the commonly used drug for treating AD in clinical practice [7]. However, the therapeutic effect of AChE inhibitor is not ideal. This drug can improve the patient’s neuropsychiatric symptoms but cannot reverse AD development. The latest research showed that AChE possibly played multiple roles in AD development because its peripheral binding site (PAS) could promote the production of Aβ protein and accelerate its deposition. Therefore, the catalytic activity site (CAS) and PAS dual-binding site inhibitor acting on AChE may exhibit multiple mechanisms of action for AD treatment. One simple way to design the dual-site AChE inhibitor is to connect two pharmacophores to a dyad in a certain manner. This dimer can simultaneously act on the CAS and PAS of AChE, improve the inhibitory activity on AChE, and inhibit AChE-induced Aβ aggregation [8,9]. This finding has become the new direction in developing new cholinesterase inhibitors. Butyrylcholinesterase (BuChE) and AChE both exist in the body of mammals. However, because BuChE mainly exists in the peripheral nervous system, BuChE inhibition will cause side effects to the peripheral nervous system [10,11]. The dose effect curves of tremor (central effects) and salivation (peripheral effect) showed that the highly selective cholinesterase inhibitor donepezil exerts better therapeutic index inhibition than tacrine, which can inhibit both AChE and BuChE [12,13]. Furthermore, recent reports showed that the activity of BuChE decreased in the nerve synapses of AD patients [14]. These results indicated that AChE inhibitors exhibiting high selectivity and high activity had excellent development prospects in the research and development of AD drugs.

**Figure 1:**
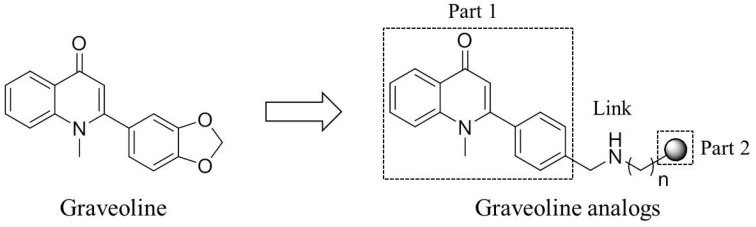
Structure of graveoline and the conventional design idea of dual-target AChE inhibitors.

The discovery of active ingredients from natural products and structural modifications are important ways of developing new drugs. Graveoline is a 2-phenyl quinolinone compound and exists in rutaceous plants. Graveoline exhibits extensive pharmacological effects, such as anti-bacteria, spasmolysis, and anti-tumor [15,16,17]. In our early efforts to search for novel AChE inhibitors, the graveoline analog 2 was identified as AChE inhibitor with an IC_50_ value of 3.65 μM. On the basis of the dual-site strategy that was designed according to the structural characteristics of graveoline and benzyl piperidine AChE inhibitors [18,19,20], we designed a dual-site AChE inhibitor that acts on both the AChE central CAS and PAS with 2-phenyl quinolinone as the parent (Figure 1). The overall strategy in molecular structure design includes the binding of the parent aromatic ring and an appropriate amino terminal side chain with the central catalytic site of AChE. We aimed to improve AChE inhibitory activity and AChE/BuChE selectivity by substituting the 4′-benzene ring in the 2-phenyl quinolinone molecule and by introducing an amino terminal side chain. Furthermore, the introduction of amine side chain could improve the water solubility of the graveoline analogs. This conceptual design was confirmed by the results of a follow-up study. 

## 2. Results and Discussion

### 2.1. Chemistry

**Scheme 1:**
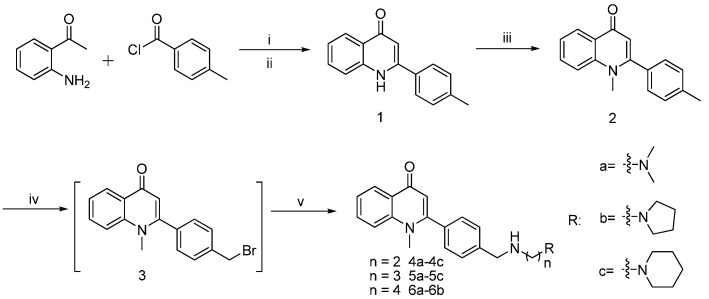
Synthesis of derivatives. Reagent: (i) 4-Rbenzoyl chloride ,THF, rt; (ii) *t*-BuOH/*t*-BuOK, 75 °C; (iii) CH_3_I/K_2_CO_3_, DMF; (iv) NBS/ benzoyl peroxide, CCl_4_, reflux; (v) RNH_2_, CH_2_Cl_2_, rt.

Scheme 1 shows the synthetic route of the compound. By reacting 2-aminoacetophenone (raw material) with benzoyl chloride to obtain amide, we obtained carbostyril midbody **1** by cyclization catalyzed by potassium tert-butoxide. To obtain the methylated product **2**, we reacted compound **1** with the alkylate reagent CH_3_I under K_2_CO_3_. Compound **2** was then mixed 10 times with CCl_4_ and then reacted with NBS to obtain bromo-product **3**; this reaction was catalyzed by benzoperoxide. Product **3** was mixed with various fatty amino to obtain the end-product fatty amino-substituted analogs **4a**–**4c**, **5a**–**5c**, and **6a**–**6b** by amination reaction.

### 2.2. Inhibitory Activity of Graveoline Analogs on AChE and BuChE

The inhibitory activity of the synthesized graveoline analogs on cholinesterase *in vitro* was studied using the Ellman method [21]. Tacrine was used as positive control. Table 1 shows the enzymatic inhibitory activity of the graveoline analogs.

**Table 1:** *In vitro* inhibition IC_50_ and selectivity index of graveoline analogs **4a**–**4c**, **5a**–**5c** and **6a**–**6b** on Acetylcholinesterase (AChE) and butyrylcholinesterase (BuChE).

Compound	R	*n*	AChE Inhibition ^a^, (IC_50_) nM	BuChE Inhibition ^b^, (IC_50_) nM	Selectivity Index ^c^
**4a**	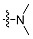	2	78.53 ± 1.76	3522 ± 46	45
**5a**		3	33.36 ± 0.78	2680 ± 32	80
**6a**		4	35.51 ± 0.96	3674 ± 39	103
**4b**	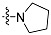	2	10.18 ± 1.33	2470 ±85	223
**5b**		3	6.47 ± 0.15	1886 ± 54	291
**6b**		4	12.64 ± 0.57	2434 ± 49	193
**4c**	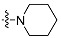	2	9.63 ± 0.76	1543 ± 29	160
**5c**		3	2.36 ± 0.24	1147 ± 31	486
**2**			3646 ± 38	9306 ± 76	3
Tacrine			224.7 ± 1.24	31.26 ± 0.34	0.1

^a^ IC_50_: 50% inhibitory concentration (means ± SEM of three experiments) of AChE; ^b^ IC_50_: 50% inhibitory concentration (means ± SEM of three experiments) of BuChE; ^c^ Selectivity Index = IC_50_ (BuChE)/IC_50_ (AChE).

Table 1 shows that all analogs, except for parent compound **2**, showed strong inhibitory ability and selectivity to AChE after the 4’-benzene ring substituted the 2-site and after introducing the side chain. The selective coefficient ranged from 45 to 486, and the IC_50_ values were expressed in the nanomole concentration level; the inhibitory activities of the analogs were better than those of tacrine. On one hand, we speculated that these side chains were protonated under physiological pH. The protonated side-chain quaternary ammonium was similar to the structure of the acetylcholine molecule, easily entered the enzyme activity pocket, and interacted with the amino acid residue in the triplet catalytic center. Moreover, the protonated side-chain quaternary ammonium can induce the π–π accumulation of the stabilized ring aromatic structure of the parent compound and the PAS of the aromatic amino acid residues, thus promoting the combination of parent ring structure and the AChE PAS. On the other hand, the alkylamino side chain can combine with the active site of the enzyme and guide the molecules into the narrow active valley of cholinesterase such that the other groups mutually combine with amino acid residues in the PAS region. BuChE contains no PAS binding site and its active center is wider than that of AChE. Thus, the introduction of the compound chain exerts a lower effect on inhibitory activity against BuChE than against AChE.

The change in the side chains can also significantly affect the inhibitory activity of the graveoline analogs on cholinesterase. Table 1 shows that if the end groups are the same, the highest inhibitory activity against AChE is exhibited by compounds **5a**, **5b**, and **5c**, whose terminal amino is linked with the parent by six chemical bonds (*n* = 3). The inhibitory activities to shorten (*n* = 2, **4a**, **4b**, and **4c**) or extend (*n* = 4, **6a**, and **6b**) the side chain of the compounds are reduced. A short side chain does not allow the terminal amino to reach the choline binding site, whereas an excessively long side chain increases the flexibility and disturbance of the total molecular structure, which was more likely to cause the side chain to not deeply reach the enzyme active valley, thereby affecting the combination of the compounds and enzymes.

The analogs bearing N heterocycle as a side-chain terminal structure displayed strong inhibitory ability (AChE). When the lengths of the side chain were the same, the compounds bearing piperidyl (**4c** and **5c**) as their terminal group displayed the strongest inhibitory activity, followed by pyrrolidyl (**4b**, **5b**, and **6b**); the compounds bearing the terminal group *N*-methyl (**4a**, **5a**, and **6a**) showed the weakest inhibitory activity. Therefore, the size of the terminal groups and the steric hindrance greatly affected the combination of compounds and enzymes. The relatively large terminal groups increased the rigidity of the entire molecular structure, thereby reducing the disturbance caused by the side chain in the enzyme active valley and increasing the binding capacity of the small molecules and enzymes. These results demonstrated the importance of the length and size of the amide side chain for strong cholinesterase inhibitory ability.

### 2.3. Kinetic Characterization of AChE Inhibition

**Figure 2:**
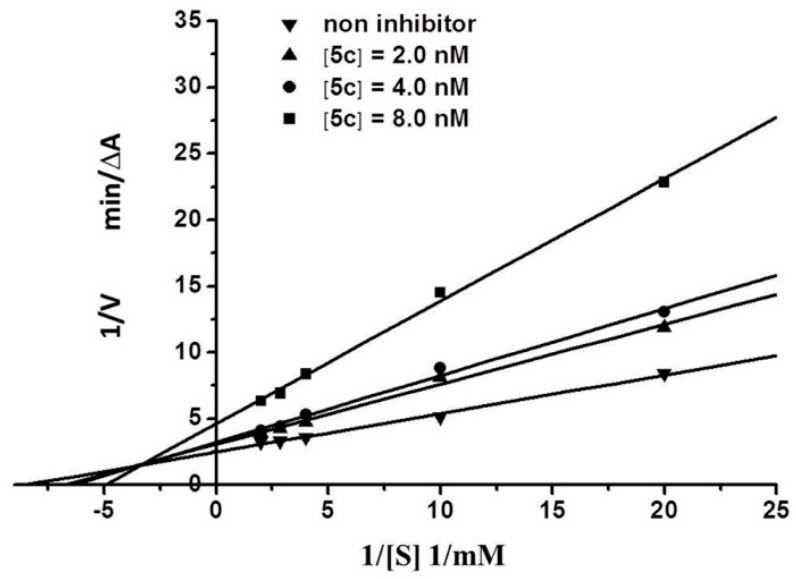
Lineweaver-Burk plot for the inhibition of AChE by **5c**.

To identify the type of inhibition exerted by the analogs on AChE, we chose compound **5c**, which exhibits the best activity, to study the inhibition kinetics of AChE. All straight lines in Figure 2 were intersected in the second quadrant of the coordinate axis, which characterizes a typical mixed inhibition. When the small-molecule ligand was combined with the AChE active center (CAS), the type of inhibition of the enzyme was classified as competitive inhibition; by contrast, when the small-molecule ligand was acted on the PAS, the enzymatic inhibition was classified as non-competitive inhibition. When the small-molecule ligand acted both on CAS and PAS, the enzymatic inhibition was classified as mixed inhibition [22]. Therefore, we speculated that the synthesized graveoline analogs interacted with the two AChE functional sites CAS and PAS; thus, the analogs showed a strong inhibitory effect. This conclusion was verified by subsequent molecular docking experiments.

### 2.4. Study on the Interaction between ***5c*** and Cholinesterase by Molecular Docking Method

**Figure 3:**
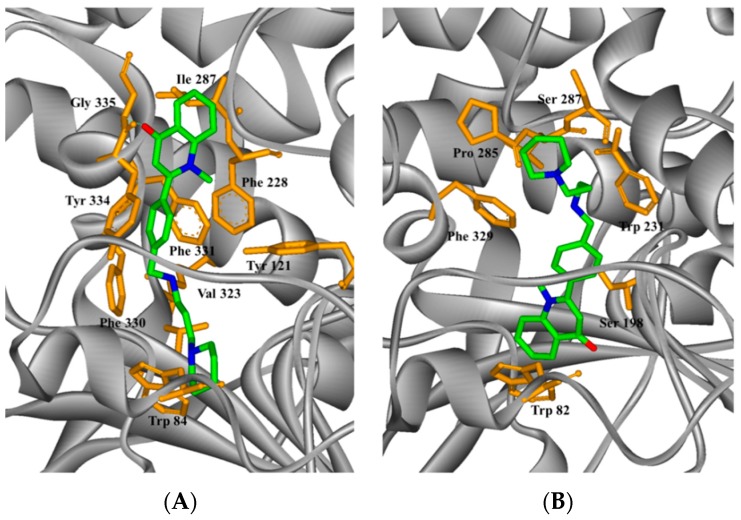
Docking models of the **5c**–enzyme complex. Representations of compound **5c** interacting with residues in the binding site of *Torpedo californica* acetylcholinesterase (TcAChE) (**A**) and human butyrylcholinesterase (HuBuChE) (**B**). The compounds are rendered in green stick models, and the amino acid residues are rendered in golden sticks.

AUTODOCK docking and PyMOL observation were used in the molecular docking between **5c**, which exhibited the strongest activity, and cholinesterase. Figure 3 shows that the optimal structural conformation of the ligand molecule **5c** was based on the docking energy value during docking between **5c** and cholinesterase. The docking result showed that the ligand molecule interacted with the functional groups of the amino acid residues found along the active valley of the AChE (Figure 3A). The parent structure of **5c** was bound to the PAS region and displayed a classic π–π stacking interaction between Tyr334, the 2-phenyl of **5c**, and Phe331, with a ring-to-ring distance of 3.6 and 3.4 Å. The carbonyl group of the quinolinone moiety established a hydrogen bond (3.7 Å) with the nitrogen atom of the main chain of Gly335. Furthermore, the conformation of the amino alkyl amine side chain matched the enzyme active valley well. The N atoms in the terminal piperidine ring were also positively charged after being solvated and formed positive ion-π interaction with the tryptophan Trp84 residue in the catalytic activity region in the bottom valley. The distance was 3.2 Å. These results showed that compound **5c** could react with both the PAS and the catalytic activity region of the enzyme, thus resulting in the strong inhibitory effect against AChE. At the same time, different interaction modes were found in **5c** in the complex with human butyrylcholinesterase (HuBuChE, PDB code: 1POI). The quinolinone moiety of **5c** reached the valley bottom with a classic π−π stacking interaction with the Trp82 residue of catalytic activity, and the ring-to-ring distance was 3.6 Å (Figure 3B). Furthermore, no obvious interaction between the ligand and the enzyme was observed. Thus, the inhibitory activity against BuChE was weak and the analogs had high selectivity.

### 2.5. AChE-Induced Aβ Aggregation: Inhibition Studies

**Figure 4:**
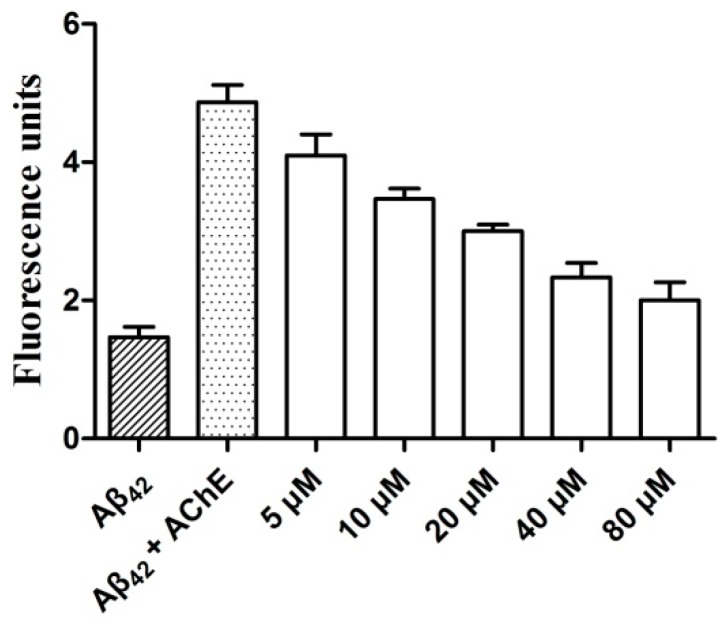
Determination of **5c** inhibition potency on AChE-induced amyloid-β (Aβ)_42_ aggregation.

To explore the combining effect of graveoline analogs on the AChE functional site PAS, compound **5c** was chosen to study the inhibitory activity of AChE-induced Aβ aggregation using thioflavin T-based fluorometric assay [8,9]. As shown in Figure 4, compound **5c** can significantly inhibit AChE-induced Aβ_42_ aggregation. The inhibitory effect of **5c** was related to the compound concentration. The combinations of the inhibitory activity of **5c** on AChE and AChE-induced Aβ aggregation indicated that the graveoline analog was a dual-site AChE inhibitor that acts on both the AChE central CAS and PAS.

### 2.6. Effects of Graveoline Analogs on Neurocyte Viability

**Figure 5:**
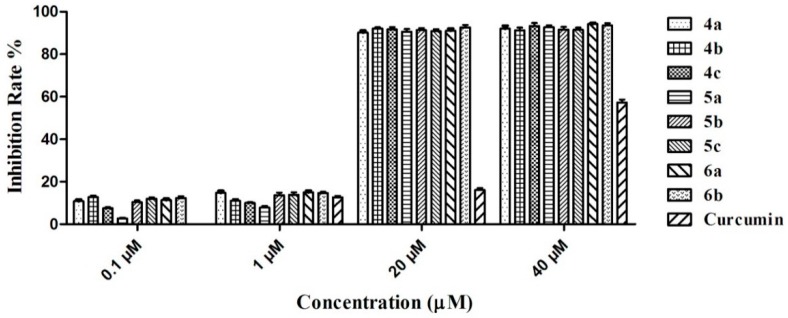
Inhibition for SH-SY5Y of compounds **4a**–**4c**, **5a**–**5c**, **6a**–**6b** and curcumin at different concentrations.

As potential anti-AD drugs, the graveoline analogs must exhibit low toxicity. Thus, we used the methyl thiazolyl tetrazolium (MTT) method [23] to perform a neurocyte (SH-SY5Y) toxicity test *in vitro*. The preliminary results (Figure 5) showed that the incubation of the SH-SY5Y cell strain with 0.1 and 1.0 μM concentrations of compounds **4a**–**4c**, **5a**–**5c**, and **6a**–**6b** for 48 h did not greatly influence the cell viability of the strain. Furthermore, the neurocyte inhibition rate was below 15%. However, cell viability was greatly influenced at 20 and 40 μM concentrations and the inhibition rates were more than 90%. By contrast, the inhibition rate of the reference substance curcumin (40 μM) on the neurocytes was 57.3%. This result showed that the synthesized compounds still exerted a certain level of cytotoxicity. However, compared with the cholinesterase activity at the nanomole level, this level of cytotoxicity is acceptable. As a drug, the blood drug concentration *in vivo* did not reach the micromole level. Cholinesterase could also be inhibited under 100 nmol or lower concentrations to prevent severe toxicity to the body.

## 3. Materials and Methods 

### 3.1. Chemistry

^1^H-NMR spectra was measured in DMSO-*d*_6_ or CDCl_3_ on a Bruker 400 NMR (Bruker, Karlsruhe, Germany) by using tetramethylsilane as internal standard. The mass spectrometer (MS) were recorded using Shimadzu LC-MS-2010A spectrometer (Shimadzu, Kyoto, Japan) with an ESI or ACPI mass selective detector. Elemental analysis was measured by the Elementar Vario EL CHNS Elemental Analyzer (Elementar, Hanau, Germany). The melting points (m.p.) were measured using the X-6 microscope melting point instrument (Beijing Tech Instrument Co. Ltd., Beijing, China), and data were not corrected. 

*2-p-Tolylquinolin-4(1H)-one* (**1**). A solution of the p-Toluoyl chloride (10 mmol) in THF (10 mL) was added to a well-stirred mixture of 2-Aminoacetophenone (10 mmol) and Et_3_N (10 mL) in anhydrous THF (150 mL) at 0 °C. After this addition, the solution was stirred for 12 h at room temperature. The precipitated solid was collected through filtration, washed with ethanol, and then dried. To mechanically stir the suspension of the dried solid in 100 mL tert-butyl alcohol, potassium tert-butoxide was added (15.0 g, 130 mmol) under N_2_ atmosphere. The reaction was heated to 75 °C for 16 h, allowed to cool to room temperature, and then washed with ice water. The precipitate was removed and recrystallized by using CH_2_Cl_2_ and methanol to give compound **1** a yellow solid in 76% yield. m.p. 169–172 °C. ^1^H-NMR (400 MHz, DMSO-*d*_6_) δ(ppm): 8.10 (d, 1H, *J* = 8.1 Hz), 7.84 (d, 1H, *J* = 8.5 Hz), 7.74 (d, 2H, *J* = 8.1 Hz), 7.65 (t, 1H, *J* = 8.0 Hz), 7.39 (d, 2H, *J* = 8.0 Hz), 7.33 (t, 1H, *J* = 8.0 Hz), 6.33 (s, 1H), 2.41 (s, 3H). APCI-MS *m*/*z*: 236.1 [M + H]^+^.

*1-Methyl-2-p-tolylquinolin-4(1H)-one* (**2**). Methyl iodide (3 mL) was added to a well-stirred mixture of the intermediate **1** (10 mmol) and K_2_CO_3_ (10 mmol) in anhydrous DMF (60 mL) at 0 °C under N_2_ atmosphere. The reaction was then allowed to warm up to 50 °C for 5 h, washed with ice water, and then eluted with CH_2_Cl_2_. The organic solvent was removed *in vacuo*, and the residue was purified using column chromatography (eluent CHCl_3_/MeOH = 40:1) to give compound **2** (0.71 g, 14%). m.p. 171–175 °C. ^1^H-NMR (400 MHz, CDCl_3_): δ 8.50 (d, *J* = 8.0 Hz, 1H), 7.71 (t, *J* = 8.0 Hz, 1H), 7.55 (d, *J* = 8.0 Hz, 1H), 7.42 (t, *J* = 7.8 Hz, 1H), 7.30 (s, 4H), 6.30 (s, 1H), 3.61 (s, 3H), 2.44 (s,3H). APCI-MS *m*/*z*: 250.1 [M + H]^+^.

#### General Procedure for the Synthesis of Graveoline Analogs (**4a**–**4c**, **5a**–**5c**, and **6a**–**6b**)

A mixture of intermediate **2** (1.8 g, 7.2 mmol), benzoyl peroxide (0.08 g), and NBS (1.56 g, 8.55 mmol) in anhydrous CCl_4_ (50 mL) were stirred at 50 °C for 5 h. After the reaction was complete, the mixture was filtered through a pad of celite and then concentrated *in vacuo*. The residue (**3**) was suspended in CH_2_Cl_2_ (5 mL), and amine (2.0 mL) was added dropwise in CH_2_Cl_2_ (5 mL). The reaction was stirred overnight and then washed with 30 mL water. Finally, the solution was extracted with CH_2_Cl_2_ (3 × 100 mL), and the combined organic layers were dried over anhydrous Na_2_SO_4_. The solvent was then removed under vacuum. The residue was chromatographed on silica gel eluting with CHCl_3_/MeOH (30:1) to obtain **4a**–**4c**, **5a**–**5c,** and **6a**–**6b** yields of 54%–73%.

*2-(4-((2-(Dimethylamino)ethylamino)methyl)phenyl)-1-methylquinolin-4(1H)-one* (**4a**). A light-yellow solid. m.p. 197–199 °C; ^1^H-NMR (400 MHz, CDCl_3_): δ 7.96 (dd, *J* = 7.5, 1.4 Hz, 1H), 7.68–7.55 (m, 3H), 7.32 (d, *J* = 7.4 Hz, 2H), 7.08 (td, *J* = 7.6, 1.6 Hz, 1H), 6.90 (dd, *J* = 7.6, 1.4 Hz, 1H), 6.85 (s, 1H), 6.45 (s, 1H), 3.92 (s, 2H), 3.52 (s, 3H), 2.63 (t, 2H), 2.54 (t, 2H), 2.32 (s, 6H); APCI-MS *m*/*z*: 336.2 [M + H]^+^; Anal. Calcd. for C_21_H_25_N_3_O. H_2_O: C, 71.36; H, 7.70; N, 11.89. Found: C, 71.28; H, 7.69; N, 11.95.

*1-Methyl-2-(4-((2-(pyrrolidin-1-yl)ethylamino)methyl)phenyl)quinolin-4(1H)-one* (**4b**). A light-yellow solid. m.p. 187–190 °C; ^1^H-NMR (400 MHz, CDCl_3_): δ 8.13 (dd, *J* = 7.5, 1.4 Hz, 1H), 7.71–7.60 (m, 3H), 7.31 (d, *J* = 7.4 Hz, 2H), 7.12 (td, *J* = 7.6, 1.6 Hz, 1H), 6.93 (dd, *J* = 7.6, 1.4 Hz, 1H), 6.86 (s, 1H), 6.45 (s, 1H), 3.92 (s, 2H), 3.52 (s, 3H), 3.03 (t, 2H), 2.64 (t, 2H), 2.56–2.43 (t, 4H), 1.66 (m, 4H). APCI-MS *m*/*z*: 362.1 [M + H]^+^. Anal. Calcd. for C_23_H_27_N_3_O. H_2_O: C, 72.79; H, 7.70; N, 11.07. Found: C, 72.83; H, 7.53; N, 11.02.

*1-Methyl-2-(4-((2-(piperidin-1-yl)ethylamino)methyl)phenyl)quinolin-4(1H)-one* (**4c**). A light-yellow solid. m.p. 185–188 °C; ^1^H-NMR (400 MHz, CDCl_3_): δ 8.04 (dd, *J* = 6.8, 1.4 Hz, 1H), 7.70–7.62 (m, 3H), 7.35 (d, *J* = 7.4 Hz, 2H), 7.14 (td, *J* = 7.4, 1.6 Hz, 1H), 6.98 (dd, *J* = 7.4, 1.4 Hz, 1H), 6.83 (s, 1H), 6.45 (s, 1H), 3.92 (s, 2H), 3.55 (s, 3H), 2.69 (t, *J* = 5.4 Hz, 2H), 2.66 (t, 2H), 2.36 (t, *J* = 5.4 Hz, 4H), 1.64 (m, 4H), 1.52 (m, 2H); APCI-MS *m*/*z*: 376.2 [M + H]^+^. Anal. Calcd. for C_24_H_29_N_3_O. 2H_2_O: C, 70.04; H, 8.08; N, 10.21. Found: C, 69.98; H, 8.15; N, 10.11.

*2-(4-((3-(Dimethylamino)propylamino)methyl)phenyl)-1-methylquinolin-4(1H)-one* (**5a**). A light-yellow solid. m.p. 182–185 °C; ^1^H-NMR (400 MHz, CDCl_3_): δ 8.17 (dd, *J* = 7.6, 1.4 Hz, 1H), 7.95–7.64 (m, 3H), 7.46 (d, *J* = 7.6 Hz, 2H), 7.22 (td, *J* = 7.6, 1.6 Hz, 1H), 7.08 (dd, *J* = 7.6, 1.4 Hz, 1H), 7.01 (s, 1H), 6.50 (s, 1H), 3.92 (s, 2H), 3.54 (s, 3H), 2.61 (t, *J* = 7.6 Hz, 2H), 2.50 (t, *J* = 7.6 Hz, 2H), 2.27 (s, 6H), 1.42 (m, 2H); APCI-MS *m*/*z*: 350.2 [M + H]^+^. Calcd. for C_22_H_27_N_3_O. H_2_O: C, 71.90; H, 7.95; N, 11.43. Found: C, 72.03; H, 7.94; N, 11.37.

*1-Methyl-2-(4-((3-(pyrrolidin-1-yl)propylamino)methyl)phenyl)quinolin-4(1H)-one* (**5b**). A light-yellow solid. m.p. 176–180 °C; ^1^H-NMR (400 MHz, CDCl_3_): δ 8.31 (dd, *J* = 7.6, 1.4 Hz, 1H), 7.89–7.64 (m, 3H), 7.48 (d, *J* = 7.6 Hz, 2H), 7.32 (td, *J* = 7.6, 1.6 Hz, 1H), 6.99 (dd, *J* = 7.6, 1.4 Hz, 1H), 6.88 (s, 1H), 6.45 (s, 1H), 3.94 (s, 2H), 3.52 (s, 3H), 3.07 (t, 2H), 2.62 (t, *J* = 7.6 Hz, 2H), 2.48 (t, 2H), 2.32 (m, 4H), 1.66 (m, 4H); APCI-MS *m*/*z*: 376.2 [M + H]^+^. Calcd. for C_24_H_29_N_3_O. 2H_2_O: C, 70.04; H, 8.08; N, 10.21. Found: C, 70.09; H, 7.99; N, 10.34.

*1-Methyl-2-(4-((3-(piperidin-1-yl)propylamino)methyl)phenyl)quinolin-4(1H)-one* (**5c**). A light-yellow solid. m.p. 177–179 °C; ^1^H-NMR (400 MHz, CDCl_3_): δ 8.26 (dd, *J* = 7.4, 1.4 Hz, 1H), 7.65 (ddd, *J* = 10.8, 8.2, 4.4 Hz, 3H), 7.32 (d, *J* = 7.5 Hz, 2H), 7.28 (td, *J* = 7.6, 1.6 Hz, 1H), 7.16 (dd, *J* = 7.6, 1.4 Hz, 1H), 6.88 (s, 1H), 6.45 (s, 1H), 3.97 (s, *2H*), 3.53 (s, 3H), 3.14 (t, *J* = 5.4 Hz, 2H), 2.60 (dd, *J* = 16.0, 7.8 Hz, 2H), 2.37 (dt, *J* = 10.7, 6.5 Hz, 4H), 1.67–1.60 (m, 6H), 1.52 (m, 2H); APCI-MS *m*/*z*: 390.2 [M + H]^+^. Calcd. for C_25_H_31_N_3_O. H_2_O: C, 73.68; H, 8.16; N, 10.31. Found: C, 73.54; H, 8.27; N, 10.26.

*2-(4-((4-(Dimethylamino)butylamino)methyl)phenyl)-1-methylquinolin-4(1H)-one* (**6a**). A light-yellow solid. m.p. 173–176 °C; ^1^H-NMR (400 MHz, CDCl3): δ 8.25 (dd, *J* = 7.6, 1.4 Hz, 1H), 7.68–7.55 (m, 3H), 7.32 (d, *J* = 7.6 Hz, 2H), 7.17 (td, *J* = 7.6, 1.4 Hz, 1H), 6.99 (dd, *J* = 7.4, 1.5 Hz, 1H), 6.85 (s, 1H), 6.50 (s, 1H), 3.95 (s, 2H), 3.54 (s, 3H), 2.73 (t, 2H), 2.62 (t, 2H), 2.39 (s, 6H), 1.76 (m, 2H), 1.63 (m, 2H); APCI-MS *m*/*z*: 364.2 [M + H]^+^. Calcd. for C_23_H_29_N_3_O. 2H_2_O: C, 69.14; H, 8.33; N, 10.52. Found: C, 69.34; H, 8.39; N, 10.47.

*1-Methyl-2-(4-((4-(pyrrolidin-1-yl)butylamino)methyl)phenyl)quinolin-4(1H)-one* (**6b**). A light-yellow solid. m.p. 169–172 °C; ^1^H-NMR (400 MHz, CDCl_3_): δ 8.21 (dd, *J* = 7.6, 1.4 Hz, 1H), 7.68–7.55 (m, 3H), 7.44 (d, *J* = 7.6 Hz, 2H), 7.23 (td, *J* = 7.6, 1.6 Hz, 1H), 7.14 (dd, *J* = 7.6, 1.4 Hz, 1H), 7.02 (s, 1H), 6.45 (s, 1H), 3.98 (s, 2H), 3.50 (s, 3H), 3.13 (t, 2H), 2.68 (t, 2H), 2.40 (t, 2H), 2.14 (m, 4H), 1.98–1.31 (m, 6H); APCI-MS *m*/*z*: 390.3 [M + H]^+^. Calcd. for C_25_H_31_N_3_O. 2H_2_O: C, 70.56; H, 8.29; N, 9.87. Found: C, 70.44; H, 8.31; N, 9.93.

### 3.2. Materials

Stock solutions of all graveoline analogs (10 mM) were prepared using DMSO and stored at −80 °C. Further dilutions to working concentrations were made with double-distilled deionized water. AChE (E.C. 3.1.1.7, obtained from electric eel), BuChE (E.C. 3.1.1.8, obtained from equine serum), 5,5′-dithiobis-(2-nitrobenzoic acid) (Ellman’s reagent, DTNB), acetylthiocholine chloride (ATC), butylthiocholine chloride, thioflavin T, and tarcine hydrochloride were purchased from Sigma-Aldrich (St. Louis, MO, USA). Aβ_42_ was purchased from Millipore (Billerica, MA, USA). 3-(4,5-Dimethylthiazol-2-yl)-2,5-diphenyltetrazolium bromide (MTT) was purchased formal Aladdin (Shanghai, China). The SH-SY5Y cell line was obtained from the Animal experimental center of Sun Yat-sen University. 

### 3.3. In Vitro Inhibition of AChE and BuChE

*In vitro* AChE assay: all methods were performed under 0.1 M KH_2_PO_4_/K_2_HPO_4_ buffer at pH 8.0 by using a Shimadzu 2450 spectrophotometer. The solutions of the enzyme were prepared to achieve 2.0 units/mL in 2 mL aliquots. The experiment system contained phosphate buffer, pH 8.0 (1 mL), 50 μL of 0.01 M substrate (ATC), 50 μL of 0.01 M DTNB, and 10 μL of enzyme. The substrate was added into the experiment system containing the DTNB, buffer, and enzyme with inhibitors after 15 min of incubation. The activity of the enzyme was determined by recording the increase in absorbance at 412 nm in 1 min intervals at 37 °C. Calculations were conducted according to the assay of Ellman *et al*. [18]. The *in vitro* BuChE assay was performed using the method described above.

The kinetic characterization of AChE was performed using the reported method [19]. Three concentrations (2, 4, and 8 nM) of the substrate were mixed in 1 mL 0.1 M KH_2_PO4/K_2_HPO_4_ buffer (pH 8.0) containing 10 μL AChE, 50 μL DTNB, and 50 μL substrate. The test compound was added into the assay solution and was pre-incubated with the AChE at 37 °C for 15 min followed by addition of the substrate. The kinetic characterization of the AChE-catalyzed ATC hydrolysis was performed spectrometrically at 412 nm. A parallel control with no inhibitor in the mixture allowed adjusting activities to be measured at various times.

### 3.4. Molecular Modeling

The crystal structure of AChE (code ID: 1EVE) and of human BuChE (code ID: 1POI) were obtained from the Protein Data Bank after eliminating the inhibitor and water molecules. The 3D structure of **5c** was built and geometry optimization was performed using molecular mechanics. Docking studies were performed using the AUTODOCK 4.0 program (The Scripps Research Institute, San Diego, CA., USA). By using the AUTODOCK 4.0 package, we adopted the hybrid Lamarckian genetic algorithm as the searching algorithm to provide the compound with full flexibility. AUTODOCK Tools was used to assign Solvation parameters and Kollman charges for all atoms in AChE and BuChE. The grid for energy evaluation was placed at the bottom of the active site gorge (AChE 2.781 64.383 67.971; BuChE 112.0 20.0 40.0) with grid points in the x-, y-, and z-axes set to 50 Å × 50 Å × 50 Å and separated by 0.375 Å. The flexible ligand docking of the compounds was also performed. The docking calculations were performed using the Lamarckian genetic algorithm, and all parameters were the same for each docking. We initially used a population of random individuals (population size: 150), with a maximum number of 2,500,000 energy evaluations. At the end of a docking procedure (150 docking runs), the resulting positions were clustered according to a root mean square criterion of 1.5 Å.

### 3.5. Thioflavin T-Based Fluorometric Assay

The self-mediated Aβ_42_ aggregation was evaluated through the thioflavin-T fluorescence assay [9]. A mixture of 230 μM Aβ_42_ peptide, 2.30 μM AChE (Aβ_42_/AChE molar ratio 100:1), and the indicated concentrations of compound 5c (5, 10, 20 ,40 and 80 μM) were added to the phosphate buffer (pH 7.40, 0.01 M). After co-incubated in 37 °C for 48 h, thioflavin-T (5 μM in 50 mM glycine-NaOH buffer, pH 8.00) was added. Fluorescence was monitored with excitation at 450 nm (λ_ex_) and 485 nm (λ_em_) by the Spectrofluorimeter LS55. The fluorescence intensity values reached at the plateau (around 300 s) were recorded after subtracting the background fluorescence. The solutions containing Aβ_42_, and Aβ_42_ plus HuAChE was checked as control. Each experiment was run in triplicate.

### 3.6. Cellular Toxicity

The SH-SY5Y cell line was seeded on 96-well plates at a density of 1.0 × 10^4^ cells/well. After overnight incubation, the cells were treated with various concentrations (0.1, 1, 20, and 40 μM) of graveoline derivatives for 48 h. Subsequently, 10 μL of 5 mg/mL MTT solution was added to each well and further incubated for 4 h at 37 °C. The solution was carefully removed, and each well was treated with dimethyl sulfoxide (DMSO) (200 μL for each well). The optical density of the wells was then read on the microplate reader at 490 nm. The parallel tests of all drug doses were performed in triplicate.

## 4. Conclusions 

This study designed and synthesized a series of new graveoline analogs on the basis of the structural characteristics of AChE dual-site inhibitors. The activity, mechanism of action, binding mode, and neurotoxicity were explored. The results showed that all analogs displayed inhibitory ability and inhibitory selectivity against AChE after 4′-benzene ring substituted the 2-site and after introducing the side chain. Compared with that of the parent compounds, the selectivity of the analogs was reversed. The structure analysis showed the best activity (**5c**) when the two sites in the graveoline parent ring substituting phenyl and amino terminal had six chemical bonds (*n* = 3), and the terminal amino among these graveoline analogs was piperidine. The enzyme kinetics, molecular docking studies, and thioflavin T-based fluorometric assay showed that these compounds could combine both with the AChE central catalytic site and PAS. Furthermore, the cytotoxicity study showed that the neurocyte (SH-SY5Y) viability was unaffected at low compound concentrations. The results indicated that these compounds offer potential applications in AD treatment.
